# Patient Journey and Treatment Patterns of Non-Muscle-Invasive Bladder Cancer in Mexico

**DOI:** 10.3390/curroncol33100597

**Published:** 2026-10-02

**Authors:** Anna Scavuzzo, Ricardo Alonso Castillejos-Molina, Iván Bravo-Castro, Miguel A. Jiménez-Ríos, José G. Campos-Salcedo, Miguel A. Jiménez-Dávila, Brenda Suaste-Carmona, Lucía E. Flores-García, Dominihemberg Ferreira, Victoria Castillo, Gabriela Lugo-Martínez, Francisco T. Rodríguez Covarrubias

**Affiliations:** 1 Department of Urological Tumors, Instituto Nacional de Cancerología (INCan), Ciudad de México 14080, Mexico; 2 Department of Urology, Instituto Nacional de Ciencias Médicas y Nutrición Salvador Zubirán (INNSZ), Ciudad de México 14080, Mexico; 3 Department of Urology, Hospital Central Militar, Secretaría de la Defensa Nacional, Ciudad de México 11600, Mexico; 4 MSD, Ciudad de México 01090, Mexico; 5 MSD, São Paulo 04583-110, Brazil; 6 TISalud, Ciudad de México 06700, Mexico

**Keywords:** NMIBC, patient journey, bladder cancer, treatment patterns

## Abstract

Non-muscle-invasive bladder cancer (NMIBC) accounts for most newly diagnosed bladder tumors, yet little is known about how it is managed in Mexico, particularly during prolonged Bacillus Calmette-Guérin (BCG) shortages. This retrospective, multicenter observational study characterized the clinical and histopathological profile, the diagnostic and treatment timelines, and the adherence to guideline-recommended care of patients with NMIBC treated at three tertiary referral hospitals in Mexico City. The records of 221 adults diagnosed between January 2010 and December 2020 were reviewed, and the risk of recurrence or progression was classified according to the European Association of Urology 2019 classification. The median age was 68 years (interquartile range [IQR] 59–75) and the male-to-female ratio was 3:1. Hematuria was the most frequent presenting symptom (83.3%). Three-quarters of patients had T1 tumors, 6.3% had concomitant carcinoma in situ, 84.6% had high-grade tumors, and 92.8% were at high risk of recurrence or progression. The median interval from symptom onset to histopathologic diagnosis was 167 days (IQR 81–329) and varied between centers. After diagnosis, the median time to second-look resection was 67 days (IQR 33.5–112) and to the first BCG instillation 92.5 days (IQR 41.8–165.5). Adjuvant intravesical therapy was administered to 66% of patients, most often BCG, although fewer than half of the BCG-treated patients (45%) completed induction, and 40% of the patients eligible for a second-look transurethral resection did not undergo it. Thirty percent of patients had no documented post-treatment surveillance. These real-world data provide a baseline description of NMIBC care in Mexican tertiary centers and identify specific, measurable gaps that may inform standardization and quality-improvement efforts in resource-constrained settings.

## Simple Summary

Bladder cancer that has not spread into the muscle of the bladder wall is usually treated by removing the tumor through the urethra and then placing medicine directly into the bladder to lower the chance that it comes back. That medicine, BCG, has been in short supply worldwide for several years, and little has been published about how these patients are cared for in Mexico. We reviewed the records of 221 people with this type of bladder cancer, most at high risk of recurrence, treated at three national referral hospitals in Mexico City over eleven years. Waiting times before diagnosis were long and differed widely between hospitals; many patients did not receive the repeat surgery or the full course of bladder medicine that guidelines recommend, and follow-up was inconsistent. These findings show where care in lower-middle-income countries, like Mexico, differs from international recommendations and point to practical steps for making it more consistent.

## 1. Introduction

Each year, bladder cancer is diagnosed in approximately 74,000 patients in the United States and more than 430,000 patients worldwide [1]. It is one of the ten most commonly diagnosed cancers in men and the eighth leading cause of cancer-related death in this group [2]. Approximately 3.0% of all new cancer diagnoses and 2.1% of all cancer deaths are associated with urinary bladder cancer [2]. Male patients have a three- to four-fold greater risk of developing urothelial bladder carcinoma during their lifetime [3]. Despite the lower incidence in women, they have a worse prognosis [3]. The incidence varies markedly across geographical regions, with the highest rates (age-standardized incidence rate per year (ASR) in males ≈ 20 per 100,000 and ASR in females ≈ 4.5 per 100,000 per year) observed in Europe and North America [2]. Worldwide differences in exposure to risk factors are largely responsible for the observed variability in occurrence; however, part of the geographical differences may also be attributable to differences in access to care and availability of diagnostic procedures [2]. Established risk factors further shape the epidemiological profile of the disease. Tobacco smoking is associated with approximately half of all cases, and the excess risk persists for years after cessation [4,5]. Body mass index has been proposed as an independent risk factor; analyses suggest a 4.2% increase in risk for every 5 kg/m^2^ above 30 kg/m^2^ [6]. Gross hematuria is the most common presenting symptom, reported in approximately 85% of cases, and the distinction between gross and microscopic hematuria is prognostically relevant: microscopic hematuria carries a 0.5–10.5% probability of malignancy, whereas gross hematuria conveys a 13–34% probability [7]. Bladder cancer can be classified into muscle invasive bladder cancer (MIBC) or NMIBC. At presentation, roughly 70–75% of patients have NMIBC, and approximately 50% of these are low grade [8]. NMIBC has a five-year recurrence rate between 31% and 78% and a progression rate between 1% and 45% [8,9].

Patients with non-muscle-invasive bladder cancer (NMIBC) can be stratified into four risk groups based on the number and size of tumors, recurrence rate, tumor stage, presence of carcinoma in situ, tumor grade, and involvement of the prostatic urethra, in order to guide treatment following the initial transurethral resection of bladder tumor (TURBT) [10,11]. Several clinical guidelines are available for the management of bladder cancer. While broadly aligned, current guidelines differ in key aspects due to variations in the underlying evidence base [10,12,13]. Typically, the management of NMIBC involves transurethral resection followed by intravesical adjuvant therapy tailored to the patient’s risk profile [13]. Bacillus Calmette-Guérin (BCG) is the first-line immune-based adjuvant therapy for patients with NMIBC at intermediate or high risk of recurrence [12].

Several international studies have documented variations in NMIBC management and adherence to guideline recommendations across different health systems [14].

Despite its status as the standard first-line therapy and its overall favorable safety profile, BCG is associated with a high rate of adverse events that can compromise patient adherence. Evidence indicates that more than 70% of patients experience some degree of side effects, and up to 8% discontinue treatment due to toxicity [15]. Around one-third of patients with NMIBC fail to respond to BCG therapy, while among initial responders, approximately 50% will develop disease recurrence or progression over time [15]. Ongoing research is focused on the development and evaluation of emerging immunotherapeutic strategies aimed at providing viable alternatives to BCG for the treatment of NMIBC [15]. Among these, pembrolizumab, an immune checkpoint inhibitor that activates the antitumor immune response by blocking Programmed Death-1 (PD-1), has been approved by the FDA for the treatment of NMIBC patients with BCG failure [16].

This study provides a comprehensive, multicenter description of the NMIBC patient journey in Mexico, integrating diagnostic timelines, treatment patterns, and follow-up strategies across national referral centers. Focused on care processes rather than on outcomes alone, these data aim to identify actionable gaps in NMIBC management and support future improvements in care pathways within the Mexican healthcare system.

## 2. Materials and Methods

This observational, descriptive, retrospective database study aimed to deepen the understanding of the clinical and histopathologic characteristics of NMIBC and of its treatment patterns in real-world settings in Mexico.

This study included all patients from the electronic database of the three participating hospitals who were diagnosed with NMIBC between 1 January 2010 and 31 December 2020 and who met the selection criteria. Risk stratification was performed according to the European Association of Urology 2019 guidelines on non-muscle-invasive bladder cancer [8]. Throughout this manuscript, risk of recurrence or progression and tumor grade are treated as distinct variables and are never used interchangeably. Tumor grade was recorded as low grade or high grade using the two-tier system retained in the current 2022 World Health Organization classification [17], a histological classification based on nuclear and architectural atypia. Risk of recurrence or progression is a composite clinical category that incorporates stage (Ta versus T1), grade, the presence of CIS, tumor size, multifocality and recurrence pattern; grade is only one of several contributing factors, so a low-grade tumor is not automatically low risk. Under the EAU 2019 classification [8], a low-grade Ta tumor still qualifies for the high-risk-of-recurrence category when it is multiple, recurrent and larger than 3 cm; a low-grade Ta tumor meeting only one of these three criteria in isolation (multifocality alone, size alone, or a single early recurrence) instead falls in the intermediate-risk category.

### 2.1. Time Interval Definitions

For the analysis of diagnostic and treatment timelines, the date of diagnosis was defined as the date of histopathologic confirmation of NMIBC recorded in the participating center’s medical record, typically corresponding to the pathology report following transurethral resection of the bladder tumor (TURBT).

Time-to-event variables were calculated as the interval in days between the date of diagnosis and the date of the corresponding intervention recorded in the database (e.g., first surgical procedure, first intravesical instillation with MMC or BCG, second surgery, and other procedures). Time to diagnosis was defined as the interval (in days) between the date of first reported urinary symptoms and the date of histopathologic confirmation of NMIBC. Post-diagnostic intervals were defined as the time from histopathologic confirmation to each subsequent intervention. Because histopathologic confirmation is issued on the specimen obtained at resection, the initial TURBT precedes the recorded diagnosis date in some patients, resulting in negative values. These values were retained in the descriptive tables for transparency, and a sensitivity analysis excluding them was performed for the diagnosis-to-first-intervention interval.

### 2.2. Statistical Analysis

A descriptive analysis was performed to characterize the study population, explore variable distributions, and identify outliers. Obvious data-entry errors were removed before creating the final analytical dataset. Continuous variables were summarized with the number of patients with valid and missing data, median, and interquartile range (IQR). Categorical variables are reported as frequencies and percentages.

For the primary objective (histopathologic characteristics, clinical patterns and treatment patterns of NMIBC), results are reported overall and by site. Time to diagnosis and post-diagnostic intervals were summarized in the same way. For the diagnosis-to-first-intervention interval, a sensitivity analysis was restricted to patients whose first TURBT was recorded on or after the date of diagnosis. Demographic characteristics for the exploratory objectives were described in the same way.

In line with the observational design and study protocol, no formal hypothesis testing, correlation analyses, or sample size/power calculations were performed; the study was intended to provide descriptive epidemiological and treatment pattern estimates only.

### 2.3. Inclusion Criteria

- Patients with a pathological diagnosis of NMIBC and at least one of the following features: carcinoma in situ (CIS/Tis), T1 stage, tumor size > 3 cm, or multifocality [8] were included in the database;
- Patients diagnosed between 1 January 2010 and 31 December 2020;
- Tumors with predominant urothelial histology (>50%).

### 2.4. Exclusion Criteria

- Patients with less than 6 months of follow-up;
- Simultaneous diagnosis of other malignancies, except non-melanoma skin cancer, other noninvasive carcinomas, or neoplasia in situ;
- Patients included in a clinical trial for the treatment of NMIBC;
- Patients previously treated for bladder cancer;
- Patients with small cell or neuroendocrine histology were excluded.

In this study we used data gathered from three tertiary care hospitals in Mexico City: Hospital Central Militar, Instituto Nacional de Ciencias Médicas y Nutrición Salvador Zubirán (INCMNSZ) and Instituto Nacional de Cancerología (INCAN), all of which are considered reference centers.

This study required review by an Institutional Review Board or Independent Ethics Committee (IRB/IEC). As the study was carried out in Mexico, approval from the corresponding IRB/IEC was required, and documented approval was obtained prior to the start of the study; approval codes and dates for each participating site are listed in the Institutional Review Board Statement.

Statistical analysis was conducted with IBM^®^ SPSS software version 30 and Microsoft^®^ Excel Version 15.

## 3. Results

A total of 221 patients were included after removal of one duplicated record. Of these, 62.4% were treated at the Instituto Nacional de Cancerología (INCAN), 24% at the Hospital Central Militar (HOMIL), and 13.6% at the Instituto Nacional de Ciencias Médicas y Nutrición “Salvador Zubirán” (INCMNSZ). The overall male-to-female ratio was 3:1. The median age at diagnosis was 68 years (IQR 59–75), with similar values at INCAN (67 years; IQR 57–75), HOMIL (67 years; IQR 59–74) and INCMNSZ (69 years; IQR 63–79).

The median body weight was 72 kg (IQR 64–80) and the median BMI 26.2 kg/m^2^ (IQR 23.8–29.3), with similar values across centers: 74 kg (IQR 66–78) and 25.9 kg/m^2^ (IQR 24.1–30.6) at INCMNSZ, 71.3 kg (IQR 60.4–80.5) and 26.3 kg/m^2^ (IQR 23.5–29.1) at INCAN, and 73 kg (IQR 66–79) and 26.1 kg/m^2^ (IQR 24.2–28.2) at HOMIL. With respect to smoking history, the overall ratio of smokers to non-smokers was 1.4:1 (59% vs. 41%).

### 3.1. Clinical Presentation

Analysis of patient distribution by clinical stage revealed that three-quarters (168 of 221) were classified as T1, whereas nearly all remaining cases were diagnosed at the Ta stage (51 patients, 23%). The predominance of T1 disease was consistent across institutions, accounting for 73% of patients at INCMNSZ, 81% at INCAN and 64% at HOMIL (Table 1).

Among these patients, 14 (6.3%) had concomitant carcinoma in situ: nine at INCAN, three at INCMNSZ and two at HOMIL. Two additional patients had isolated carcinoma in situ (Tis), one at INCAN and one at INCMNSZ. Hematuria was the most common presenting symptom, recorded in 184 patients (83.3%) (Table 1).

Among the 144 patients with documented tumor number, single lesions were more frequent than multiple lesions (81 [56%] vs. 63 [44%]) (Table 2).

Most tumors were high grade (187 patients, 84.6%), including 26 of 30 at INCMNSZ (86.7%) and 137 of 138 at INCAN (99.3%), whereas low-grade tumors predominated at HOMIL (29 of 53, 54.7%). Under the EAU 2019 classification, 205 patients (92.8%) were at high risk of recurrence or progression, 7 (3.2%) at intermediate risk and 9 (4.1%) at low risk. The nine low-risk patients, all treated at HOMIL, had a single low-grade Ta tumor smaller than 3 cm without CIS and therefore did not meet the inclusion criteria on the recorded variables; they were retained in all analyses. Lymphovascular invasion was documented in only five cases (2.26%): two each at HOMIL and INCMNSZ and one at INCAN.

Histological subtype information was unavailable in a substantial proportion of patients, and tumor number and size were not uniformly documented across all records. Consequently, the prevalence of variant histologies is likely underestimated, and multifocality could be reliably assessed only in a subset of cases. These gaps may affect the precision of risk stratification and must be considered when interpreting comparisons between institutions.

Data on histological variants were incomplete; among the cases with available information, the most prevalent subtype was papillary carcinoma, accounting for 33.9% of all cases and 79% of those with an identified pathological diagnosis. At INCAN, squamous and glandular carcinomas were found in 5.8% and 3.6% of patients (eight and five patients, respectively), representing 3.6% and 2.3% of the overall cohort. At this center, these subtypes were more common than papillary carcinoma, which, like the ring cell variant, accounted for 2.9% of INCAN patients (4 cases each). Plasmacytoid carcinoma followed in frequency, with two reported cases (1.4%). The least common subtypes—sarcomatoid, epidermoid, and giant cell carcinoma—each had a single identified case, representing 0.72% of their respective institutional cohorts and 0.45% of all patients.

In four INCAN cases, two histological variants were identified in the same patient: one with micropapillary and glandular features, one with glandular and ring cell components, another with sarcomatoid and squamous elements, and the last with both glandular and squamous differentiation. Thus, the glandular subtype was the most frequently co-occurring histological variant.

### 3.2. Time to Diagnosis and Treatment

The median time from symptom onset to histopathologic diagnosis was 167 days (IQR 81–329; *n* = 221). It was longest at INCAN (228 days; IQR 102–378) and shorter at INCMNSZ (150 days; IQR 60.5–248.5) and HOMIL (125 days; IQR 76–185).

The initial TURBT was recorded before the diagnosis date in 175 of the 209 patients with an evaluable TURBT date (84%), with a median interval of −25 days (IQR −54 to −5). Under the index date used, the diagnosis-to-first-intervention interval therefore reflects mainly the time between resection and the issuing of the pathology report, together with resections performed at referring institutions, rather than a treatment delay. The pattern was consistent across centers (INCMNSZ −2 days, IQR −5.5 to 0; INCAN −36 days, IQR −76 to −6; HOMIL −25 days, IQR −41 to −5). After excluding the 175 patients whose first TURBT preceded the recorded diagnosis date, the median interval in the remaining 34 patients (15 at INCMNSZ, eight at INCAN and 11 at HOMIL) was 8 days (IQR 1–92.8). This subgroup is small and atypical: at INCMNSZ, eight of the 15 patients had the resection and the diagnosis recorded on the same day, and the INCAN estimate rests on eight patients.

Intervals to post-diagnostic interventions were clinically more informative. The median time from diagnosis to second-look TURBT was 67 days (IQR 33.5–112), ranging from 42.5 days (IQR 28.8–78.8) at INCMNSZ to 70 days (IQR 42–112) at INCAN. The median time to the first BCG instillation was 92.5 days (IQR 41.8–165.5), shorter at HOMIL (49 days, IQR 16–96) than at INCMNSZ (117 days, IQR 54.5–208.5) and INCAN (114.5 days, IQR 53.5–173.5). The 17 patients who underwent subsequent radical surgery did so a median of 406 days (IQR 152–1085) after diagnosis.

The median time from diagnosis to the first adjuvant instillation of either agent was 77 days (IQR 19.5–139.5; *n* = 147). The first MMC dose was recorded a median of 24 days before the diagnosis date (IQR −44.8 to −16.5; *n* = 22), which may reflect instillations administered in the peri-resection period before the pathology report was issued; 18 of these 22 patients were treated at HOMIL. Estimates for MMC at INCMNSZ (*n* = 3) and INCAN (*n* = 1), and for subsequent surgery at HOMIL (*n* = 1), rest on very few patients and are reported for completeness only (Table 3).

### 3.3. Treatment Strategies

Slightly more than half of the population (58%) required a second transurethral intervention, with all the institutions showing a similar pattern (57%, 58% and 60% for INCAN, HOMIL and INCMNSZ, respectively). Only 8% of patients underwent an additional surgical procedure, such as radical cystectomy, a figure in line with the average for patients under similar conditions at INCAN. By comparison, INCMNSZ performed the highest proportion of subsequent surgeries (17%), whereas HOMIL performed the lowest (2%).

Table 4 details the distribution of subsequent surgical techniques after the initial transurethral intervention at each institution. These include radical cystoprostatectomy (RCP), radical cystectomy with hysterectomy (RCH), pelvic exenteration (PE), bilateral lymph node dissection (BLND), ureterostomy (U), bilateral ureterostomy (BU), and nephroureterectomy plus vaginal resection (NUR + VR).

### 3.4. Induction and Maintenance Adjuvant Therapy

Sixty-six percent of patients received some form of adjuvant local therapy, with 87% treated with BCG and the remaining 13% receiving mitomycin C (MMC). Nearly half of the BCG-treated patients (45%) completed the induction phase, whereas none of the MMC-treated patients achieved this outcome. Both therapies were administered to only four patients: two from the INCAN cohort and one from each of the other institutions.

Half of the patients entered a maintenance phase with either agent, although none received both agents. BCG was the preferred therapy in 98% of patients, with two-thirds of these patients completing maintenance therapy. Only two patients (both from INCMNSZ) received MMC for maintenance, and neither completed the treatment.

At INCMNSZ, the proportion of patients receiving adjuvant therapy (two-thirds) was similar to that of the overall cohort, whereas all patients at HOMIL underwent adjuvant treatment, compared with half of the patients at INCAN. Approximately 50% of the patients at INCMNSZ and INCAN completed BCG induction, as well as one-third of the patients at HOMIL. The proportion of patients receiving MMC induction was highest at HOMIL (36%), while two cases were reported at each of the other institutions.

### 3.5. Distribution of Procedures Performed According to Histological Subtype

Among patients with an unspecified histologic subtype, nearly 60% required a second-look procedure, whereas 93% did not meet the criteria for further surgical intervention. This distribution mirrored that of patients diagnosed with papillary bladder cancer, comprising 75 patients—44 who required a second TURBT and 31 who did not, with 91% not meeting the criteria for additional surgery.

In contrast, 50% of patients with squamous cell carcinoma (three of six) underwent a second TURBT, and only one required radical cystoprostatectomy with urinary diversion via Studer’s ileostomy. Among the less common histological subtypes, two of three adenocarcinoma cases (66.6%) required a second TURBT, a pattern also observed in sarcomatoid, epidermoid, and giant cell carcinomas (one case each). No additional procedures were performed in patients with ring cell or plasmacytoid carcinoma. Among the four patients with coexisting histological variants, three required a re-TURBT, but none underwent further surgery; thus, these patients were not included in the corresponding tables.

The proportions reported below were obtained in a histology-stratified tabulation of adjuvant therapy. Because they are calculated within each histological subgroup, they are not additive with, and should not be compared directly to, the overall induction-completion rate of 45%, which was derived from the primary analysis of all BCG-treated patients and is the reference estimate for the cohort. Of the 126 patients with an unspecified histological subtype, 60% received adjuvant treatment. Among them, only three underwent mitomycin induction, with just one completing the regimen. The remaining patients received BCG, achieving an 88% completion rate. Additionally, two patients received both therapies. Maintenance therapy was administered to 59 patients (47%), all of whom received BCG, for a 78% completion rate.

For papillary carcinoma patients, 85% received adjuvant therapy; one-third were treated with mitomycin, although none completed the regimen, while the remaining two-thirds received BCG, with a 58% completion rate. Two patients received both therapies. Maintenance therapy was given to 57% of these patients; only two patients received mitomycin, while the remaining patients were treated with BCG, half of whom completed the regimen.

For patients with squamous and glandular carcinoma, two patients received both induction and maintenance therapy, exclusively with BCG. This accounted for 33% of the squamous carcinoma cases and 66% of the glandular carcinoma cases. All completed induction, but only half completed maintenance.

Among the three patients with ring cell carcinoma, only one underwent both induction and maintenance with BCG, successfully completing both. The same was observed in the single cases of epidermoid and giant cell carcinoma, as well as in the only patient (25%) with two simultaneous histological subtypes who received adjuvant therapy. In contrast, none of the patients with sarcomatoid or plasmacytoid carcinoma received adjuvant treatment.

### 3.6. Follow-Up Strategies

Cystoscopy was preferred over cytology as a follow-up strategy at an approximately 2:1 ratio (150 vs. 83 patients). Overall, the intensity and modality of follow-up differed across institutions. Cystoscopy was the predominant surveillance tool, but its use ranged from exclusive reliance on cystoscopy at one center to more frequent combinations of cystoscopy and cytology at another. In total, 77 patients underwent both cystoscopy and cytology during follow-up, whereas 66 of 221 patients (approximately 30%) had no documented post-treatment surveillance in the database (Table 5), and were considered lost to follow-up. These differences suggest substantial heterogeneity in follow-up strategies and in the completeness of follow-up documentation between centers. At HOMIL, follow-up was based exclusively on cystoscopy, whereas INCMNSZ showed the highest proportion of patients monitored with both cystoscopy and cytology. INCAN displayed intermediate patterns. The proportion of patients without any recorded surveillance differed markedly: none at HOMIL, eight of 30 (27%) at INCMNSZ and 58 of 138 (42%) at INCAN.

Among patients with papillary carcinoma, 10% underwent both modalities and one-quarter had no follow-up; 88% of those monitored were followed with cystoscopy. Of the three patients with glandular carcinoma, one had no surveillance, one was monitored with both modalities, and one exclusively with cystoscopy. Of the three cases of signet ring cell carcinoma, one lacked follow-up, whereas two underwent both cystoscopy and cytology.

## 4. Discussion

This study offers a detailed real-world characterization of NMIBC management in Mexico to date, focusing on the patient pathway from diagnosis through treatment and follow-up across three distinct public institutional settings, rather than on clinical outcomes. Oncological endpoints are not reported because post-treatment surveillance at the participating institutions was inconsistent and incompletely documented, with 66 of 221 patients (30%) having no recorded follow-up encounter after initial treatment. Under these conditions, any recurrence-free, progression-free or cystectomy-free survival estimate would have been derived from a non-random subset of patients whose follow-up happened to be captured, and would have been biased to an unknown degree and direction. The value of this work lies instead in a multi-institutional description of how NMIBC is managed in Mexican national referral centers, measured against the guideline-defined standard of care. Documented variation in time to diagnosis, second-look TURBT, intravesical therapy and surveillance constitutes an interpretable description of care delivery in its own right, particularly because descriptions from Latin American samples are limited. Because the resource constraints described here, notably the prolonged BCG shortage, incomplete pathology reporting and geographically driven loss to follow-up, are shared by many low- and middle-income settings, these data provide a baseline that may be applicable well beyond Mexico.

The demographic profile of this cohort was broadly consistent with international series: approximately 75% of patients were men, matching GLOBOCAN [3], with reported regional ratios reaching 6:1 elsewhere [18], and 59% were current or former smokers, rising to 77% at INCMNSZ. Sex distribution and smoking prevalence therefore require no special explanation and are not the findings of interest here.

More notable was the age at diagnosis. The median age of 68 years (IQR 59–75) was approximately five years below the median of about 73 years reported for Europe and the United States [13], and was similar across centers (67 to 69 years). A younger age at presentation in a referral population is compatible either with a genuinely earlier age distribution or with preferential referral of younger patients to national centers, and the present design cannot distinguish between these possibilities.

Anthropometric variables were unremarkable (median weight 72 kg and median BMI 26.2 kg/m^2^) and are reported for completeness rather than as a finding.

Most patients resided in central metropolitan areas (Mexico City and Estado de México), reflecting the concentration of hospitals in these regions. Other states, mainly in the Bajío and Pacific areas, had lower representation. Puebla and Guerrero had slightly higher representation in HOMIL than in other institutions.

Hematuria was the presenting symptom in 83.3% of patients, closely matching international reports [7]. The database did not distinguish gross from microscopic hematuria, a distinction with direct bearing on the probability of malignancy at presentation; its absence limits inference about referral thresholds and represents a documentation gap worth correcting prospectively.

Bladder cancer is typically diagnosed at an early stage, with 75% of cases not invading beyond the muscular layer [8]. However, our sample revealed a reverse distribution (76% T1, 24% Ta or Tis) [19,20].

Several tumor characteristics are associated with the risk of recurrence or progression, including lymphovascular invasion, variant histology, carcinoma in situ, tumor size of 3 cm or more, and multifocality, and patients at high risk of recurrence are recommended to receive BCG rather than mitomycin C (MMC) as first-line adjuvant therapy [12]. Under the EAU 2019 classification, 205 patients (92.8%) were at high risk of recurrence or progression, consistent with the high proportions of T1 (76%) and high-grade (84.6%) tumors, each of which places a tumor in the high-risk category [8].

Of the 205 patients at high risk of recurrence, 125 (61%) received BCG as recommended and 80 did not; MMC was used as an alternative in seven of these 80 patients. Of the 16 patients at intermediate or low risk, 12 received MMC and three received BCG.

Deviations from guideline recommendations may reflect the BCG shortage or institutional availability, although the database does not record the reasons.

The distribution of tumor size and multifocality was generally balanced, except at INCMNSZ, where multifocal disease was more frequent. These differences raise questions about institutional diagnostic practices and reporting standards.

Reasons for treatment discontinuation were not always documented but may include loss to follow-up (28 cases), progression, or intolerance. Geographic factors likely played a role, as 60% of lost-to-follow-up patients resided outside Mexico City.

A second TURBT is recommended for incomplete resections, T1 stage, high-grade tumors, large Ta lesions (≥3 cm), or multifocality [12]. In this cohort, 127 patients underwent a second-look TURBT, more than 90% of whom met the established criteria. However, 40% of eligible patients did not undergo this intervention, possibly because of loss to follow-up, disease progression, or physician discretion.

Of the 17 patients who required subsequent surgery, 76% (13/17) underwent radical cystoprostatectomy; most of these surgeries were performed at INCAN. Across the whole cohort, seven bilateral lymphadenectomies were documented; the true frequency is probably higher, as this procedure is generally included in radical surgery [21].

Urinary diversion was frequently achieved through an ileal conduit, reflecting patient suitability and institutional practices. At INCAN, three cases of bladder perforation after TURBT were reported, one of which had a fatal outcome.

The median time from symptom onset to diagnosis was 167 days, with marked institutional variability, from 125 days at HOMIL to 228 days at INCAN. Delays at INCAN may be influenced by its larger patient volume and by limitations in primary care referral pathways.

These intervals compare unfavorably with international data. A Spanish multicenter cohort reported a median of 104 days from first consultation to diagnosis [22], and an Australian series reported 69.5 days from first hematuria to diagnosis [23]. Because these benchmarks are measured from first medical contact rather than from symptom onset, they are not strictly equivalent to our measure; nevertheless, a median of 167 days, with one quarter of patients waiting more than 329 days, points to delays in the pre-diagnostic phase of care, where recognition and referral of hematuria in primary care are the most plausible targets for improvement.

After diagnosis, the median time to second-look TURBT was 67 days, beyond the two to six weeks recommended for re-resection [8], and the median time to the first BCG instillation was 92.5 days, with a two-fold difference between HOMIL (49 days) and the two other centers (114.5 to 117 days). These post-diagnostic intervals, rather than the diagnosis-to-first-intervention interval, are the clinically interpretable measures of treatment timeliness in this dataset, because the latter was negative in 84% of patients as a consequence of the index date. In the context of the prolonged BCG shortage during the study period, the delay to the first instillation is a plausible and measurable consequence of supply constraints, although the present data cannot separate supply from scheduling factors.

Cystoscopy was the predominant follow-up method, with a 2:1 ratio over cytology. At HOMIL, only cystoscopy was used, whereas INCMNSZ had the highest proportion of combined approaches (67%). Thirty percent of patients had no recorded surveillance, which hinders the evaluation of long-term outcomes and adherence to surveillance protocols.

### 4.1. Implications for Practice and Policy

Our findings suggest several priorities for improving NMIBC care in Mexico. First, the marked diagnostic and treatment delays highlight the need for early detection initiatives and more efficient referral pathways from primary care. Second, the variability in second-look TURBT, intravesical therapy use, and surveillance supports the development of standardized national NMIBC protocols and quality indicators. Third, ongoing BCG shortages require transparent, evidence-based prioritization and structured use of alternatives such as MMC where appropriate. Addressing these issues will be essential to align real-world practice with guideline recommendations within the constraints of the Mexican healthcare system.

### 4.2. Study Limitations

Although standardized data collection tools were used, some variables were missing or inconsistently recorded, reflecting real-world clinical documentation practices over an eleven-year period. Variables required for complete risk stratification were among these: histological subtype was unavailable in 57% of records, and tumor number and size were not documented in 77 (35%) and 79 (36%) records, respectively, almost all from INCAN. Comparisons of risk composition between institutions are therefore subject to differential missingness and should be read as descriptive rather than inferential. In addition, nine patients (4.1%), all from HOMIL, had low-risk disease under the EAU 2019 classification and did not meet the inclusion criteria on the recorded variables; they were retained to avoid post hoc exclusion.

Second, the inclusion of data from three distinct tertiary care institutions introduces inherent heterogeneity in clinical workflows, diagnostic approaches, and treatment decision-making. While this variability strengthens the external validity of the findings by capturing routine practice across different settings, it may also contribute to differences in data availability and reporting across centers.

Third, as this was a secondary database study, the analysis was restricted to variables predefined at the time of data collection, and no oncological outcome, including recurrence-free, progression-free and cystectomy-free survival, could be estimated. As a result, certain potentially relevant clinical details—such as reasons for treatment discontinuation, patient preferences, or physician-level decision-making factors—could not be systematically assessed.

Finally, the study population was derived from national referral centers in Mexico City, which may limit the generalizability of the findings to other healthcare settings, particularly community hospitals or regions with different resource availability. In addition, 66 of 221 patients (30%) had no documented post-treatment surveillance. This loss to follow-up precludes survival analysis, constrains any inference about long-term treatment adherence, and should be borne in mind wherever follow-up proportions are reported above. Nevertheless, these centers manage a high volume of complex NMIBC cases, and the data provide a meaningful real-world baseline for understanding care pathways for NMIBC within the Mexican healthcare system.

## 5. Conclusions

This multicenter study documents how NMIBC, predominantly at high risk of recurrence, was managed in three Mexican national referral centers over eleven years. Several gaps are specific and measurable: a median of 167 days from symptom onset to diagnosis; second-look resection performed a median of 67 days after diagnosis and omitted in 40% of eligible patients; BCG started a median of 92.5 days after diagnosis and given to 61% of patients at high risk of recurrence; and no documented surveillance in 30% of patients. The younger-than-expected median age at diagnosis and the high prevalence of T1 tumors suggest possible delays in early detection or population-specific risk factors. The histological profile observed in this cohort also differs from global reports and warrants further study.

Treatment adherence was heterogeneous in this cohort, with several patients receiving therapies that diverge from international guidelines. In the Mexican context, this may be attributable to barriers such as the limited availability of intravesical therapies, restricted access to high-cost systemic agents, variability in surgical infrastructure, and disparities in referral pathways. The low rate of second-look TURBT in eligible patients and the variability in surgical decision-making indicate the need for standardized protocols.

Future research should explore the impact of diagnostic delays, the role of second-look TURBT in preventing progression, and factors influencing adherence to adjuvant therapy. Additionally, addressing geographic disparities in follow-up and improving documentation of histopathological findings, treatment rationales, and long-term outcomes will be crucial for optimizing bladder cancer management.

Beyond describing current practice patterns, these findings provide a pragmatic framework to guide future quality-improvement strategies, institutional benchmarking, and prospective research aimed at optimizing NMIBC care pathways in Mexico and similar resource-constrained settings. Future prospective studies and registry initiatives should prioritize standardized outcome collection to evaluate the true clinical impact of the variability in NMIBC care observed here by including oncologic outcomes such as recurrence, progression, and cystectomy-free survival.

## Figures and Tables

**Table 1:** Presence of hematuria and clinical stage, overall and by center.

*n* (%)	Hematuria	Clinical Stage			Concomitant
		Ta	T1	Tis	CIS
Total (*n* = 221)	184 (83.3)	51 (23.1)	168 (76.0)	2 (0.9)	14 (6.3)
INCMNSZ (*n* = 30)	25 (83.3)	7 (23.3)	22 (73.3)	1 (3.3)	3 (10.0)
INCAN (*n* = 138)	116 (84.1)	25 (18.1)	112 (81.2)	1 (0.7)	9 (6.5)
HOMIL (*n* = 53)	43 (81.1)	19 (35.8)	34 (64.2)	0	2 (3.8)

**Table 2:** Distribution by size and number of tumors, overall and by center. Percentages are calculated over the whole cohort for the Total row and over each center’s number of patients for the center rows.

*n*	Number of Tumors		Size	
	1	>1	≤3 cm	>3 cm
Total	81 (56.3)	63 (43.8)	78 (54.9)	64 (45.1)
INCMNSZ	12 (41.4)	17 (58.6)	14 (46.7)	16 (53.3)
INCAN	38 (57.6)	28 (42.4)	27 (45.8)	32 (54.2)
HOMIL	31 (63.3)	18 (36.7)	37 (69.8)	16 (30.2)

**Table 3:** Time intervals (days) to diagnosis and to subsequent interventions, overall and by hospital center, expressed as median [Q1, Q3].

Median [Q1, Q3]	Diagnosis	First Intervention (All Values)	First Intervention, Values ≥ 0 Only (Sensitivity Analysis)	First Adjuvant Treatment	First BCG Dose	First MMC Dose	Second Look (Re-TURBT)	Second Surgery
GLOBAL (*n* = 221)	167 [81, 329]	−25 [−54, −5]	8 [1, 92.8]	77 [19.5, 139.5]	92.5 [41.8, 165.5]	−24 [−44.8, −16.5]	67 [33.5, 112]	406 [152, 1085]
INCMNSZ (*n* = 30)	150 [60.5, 248.5]	−2 [−5.5, 0]	0 [0, 58.5]	96 [43.5, 175.8]	117 [54.5, 208.5]	119 [60.5, 446.5]	42.5 [28.8, 78.8]	530 [406, 1764]
INCAN (*n* = 138)	228 [102, 378]	−36 [−76, −6]	117.5 [71.8, 283.3]	114.5 [53.5, 172]	114.5 [53.5, 173.5]	140 *	70 [42, 112]	254 [145.5, 758]
HOMIL (*n* = 53)	125 [76, 185]	−25 [−41, −5]	8 [4, 13]	11 [−29, 80]	49 [16, 96]	−30 [−50.3, −20.3]	64 [32.5, 104]	1602 *

* Value from a single patient; quartiles not applicable.

**Table 4:** Distribution of subsequent surgeries (global and by hospital center), by components of the surgical technique. Subsequent surgery is expressed as a percentage of each center’s patients, and surgical components as a percentage of patients who underwent subsequent surgery; components are not mutually exclusive.

*n* (%)	221 (100)	30 (13.6)	138 (62.4)	53 (24.0)
Subsequent surgery	17 (7.7)	5 (16.7)	11 (8.0)	1 (1.9)
	Total	INCMNSZ	INCAN	HOMIL
Radical cystoprostatectomy	13 (76)	2 (40)	10 (91)	1 (100)
Studer neobladder	2 (12)	0	2 (18)	0
Bricker conduit	9 (53)	2 (40)	7 (64)	0
Bilateral ureterostomy	1 (6)	0	1 (9)	0
Bilateral Lymph Node Dissection	7 (41)	3 (60)	4 (36)	0
Ureterostomy	1 (6)	0	1 (9)	0
Pelvic exenteration	2 (12)	1 (20)	1 (9)	0
Nephroureterectomy + vaginal resection	2 (12)	2 (40)	0	0
Radical cystectomy with hysterectomy	1 (6)	1 (20)	0	0

**Table 5:** Distribution of the number of surveillance cystoscopies and cytologies performed by patients throughout follow up.

	Cytologic Studies	Cystoscopies
TOTAL	5 [2, 9]	11 [7, 14]
INCMNSZ	4.5 [2, 9]	5 [3, 8]
INCAN	6 [2, 10]	11 [6, 16]
HOMIL	NA *	11 [10, 13]

* No cytology tests were recorded at HOMIL during the study period.

## Data Availability

The data presented in this study are available on request from the corresponding author due to hospitals’ policies.

## Abbreviations

The following abbreviations are used in this manuscript:

NMIBC	Non-muscle-invasive bladder cancer
MIBC	Muscle invasive bladder cancer
BCG	Bacillus Calmette-Guérin
INCMNSZ	Instituto Nacional de Ciencias Médicas y Nutrición “Salvador Zubirán”
INCAN	Instituto Nacional de Cancerología
HOMIL	Hospital Central Militar
ASR	Age Standardized Incidence Rate per year
TURBT	Transurethral resection of bladder tumor
PD-1	Programmed Death-1
IECs/IRBs	Institutional Review Board/Independent Ethics Committee
CIS	Carcinoma in situ
LI	Lymphovascular invasion
